# Effects of melatonin prolonged-release on both sleep and motor symptoms in Parkinson’s disease: a preliminary evidence

**DOI:** 10.1007/s10072-022-06111-x

**Published:** 2022-05-10

**Authors:** Claudio Liguori, Mariana Fernandes, Rocco Cerroni, Raffaella Ludovisi, Nicola Biagio Mercuri, Alessandro Stefani, Mariangela Pierantozzi

**Affiliations:** 1 Neurology Unit, Department of Systems Medicine, University of Rome “Tor Vergata”, Rome, Italy; 2 Sleep Medicine Centre, Department of Systems Medicine, University of Rome “Tor Vergata”, Rome, Italy; 3 UOSD Parkinson’s Disease, Department of Systems Medicine, University of Rome “Tor Vergata”, Rome, Italy; 4 IRCCS Santa Lucia Foundation, Rome, Italy

**Keywords:** Insomnia, Daytime sleepiness, Polysomnography, Motor symptoms, Movement disorder, Melatonin

## Abstract

**Background:**

Sleep-related symptoms, especially insomnia, are frequently reported by patients with Parkinson’s disease (PD) and can markedly affect motor symptoms and impair patients’ quality of life. Melatonin has been shown to improve sleep in PD patients. This pilot study aimed at evaluating the effects of a 3-month treatment with 2 mg melatonin prolonged-release (PR) on sleep and motor disability in PD patients.

**Materials and methods:**

Twelve PD patients under stable antiparkinsonian treatment were enrolled in the study. Before treatment (T0), motor dysfunction was assessed with Unified Parkinson’s Disease Rating Scale (UPDRS-III) and sleep architecture with polysomnography. Subjective sleep quality was also assessed through Pittsburgh Sleep Quality Index (PSQI) and daytime somnolence with Epworth Sleepiness Scale (ESS). Patients then started melatonin PR and all measures were repeated at the end of treatment after 3 months (T1).

**Results:**

Sleep latency significantly decreased from T0 to T1, but no other significant differences were found in PSG parameters. Melatonin PR treatment significantly reduced the ESS scores from T0 to T1, while the PSQI scores presented a trend of improvement from T0 to T1. Motor dysfunction was not improved by melatonin PR, although there was a trend in decreasing UPDRS-III. Both clinical global improvement and patient clinical global impression documented an improvement in insomnia symptoms at T1.

**Conclusions:**

These findings suggest that melatonin may improve sleep symptoms in PD patients, although further evidence is needed in larger controlled studies to confirm these results and explore the possible direct and indirect influence of sleep improvement on motor dysfunction.

## Introduction

Sleep problems are recognized in more than 60% of patients with Parkinson’s disease (PD) and have been related to a worse quality of life (QoL) and greater PD non-motor symptoms (NMS) burden [1, 2]. Degenerative processes in the sleep–wake regulatory brainstem centers and hypothalamic nuclei have been proposed as possible causes of sleep-related symptoms in PD [3].

Among sleep-related symptoms, insomnia is considered a common NMS in PD [4], which can markedly affect motor symptoms and impair patients’ QoL [2, 4]. Moreover, in patients with PD, insomnia has been described more frequently than other sleep disorders and can appear concomitantly with excessive daytime sleepiness (EDS) or rapid eye movement (REM) sleep behavior disorder [4, 5]. Moreover, insomnia in PD may also cause EDS in a bidirectional relation or even represents a part of a circadian sleep–wake rhythm dysregulation [4]. The typical manifestations of insomnia in PD patients are sleep-onset insomnia, sleep fragmentation, and early awakening in the morning, which may be triggered by the nocturnal worsening of motor symptoms, their re-emergence in the nighttime, and the wearing off phenomenon in motor fluctuating PD patients [6].

In PD, the risk factors for insomnia include the co-existence of other NMS such as depression, anxiety, and cognitive impairment, the use of dopamine agonists, the severity of motor impairment, and long disease duration [7, 8]. However, insomnia can also happen in the earliest stages of the disease in untreated patients [9] and thus increase in frequency and severity once dopaminergic treatment is started.

Insomnia treatment in PD patients is still challenging since validated drugs have not yet been approved, although long-acting dopamine agonists, such as rotigotine, proved to be useful for treating PD sleep-related symptoms [10, 11]. Considering the unsuccessful trials with non-benzodiazepine hypnotics and the unrecommended long-lasting use of benzodiazepine in PD [12, 13], melatonin may represent a newsworthy candidate to treat insomnia in patients with PD [14]. In this framework, clinical studies showed the effectiveness of melatonin, also in the prolonged-release (PR) formulation, in the treatment of insomnia in patients with PD [14, 15]. Furthermore, experimental studies suggested the positive effect of melatonin on neurodegenerative processes in PD animal models [16–20], whereas more recent clinical data propose a favorable influence of melatonin on PD NMS [14, 15, 21]. Although there are reports of PD patients experiencing a beneficial effect of sleep on motor mobility, the efficacy of melatonin on motor symptoms is still under debate [14, 22].

Despite the high prevalence and detrimental effect of insomnia in PD, this line of research was not extended to identify treatments to improve sleep. Therefore, considering the proposed effects of melatonin on sleep and motor symptoms in PD, the present pilot study aimed at evaluating the effects of a 3-month treatment with 2 mg melatonin PR on sleep and motor disability in PD patients.

## Methods

This was a prospective observational study to determine the efficacy of melatonin PR on nocturnal polysomnography (PSG)-derived sleep parameters, subjective sleep quality, and motor disability in patients with PD complaining of insomnia symptoms.

### Participants and study design

In the present study, PD patients were recruited from the PD Centre of the University Hospital of Rome “Tor Vergata”. Eligible patients had a diagnosis of idiopathic PD according to the Movement Disorder Society [23], and a Pittsburgh Sleep Quality Index (PSQI) score ≥ 5 [24]. Patients were also required to meet the following inclusion criteria: (1) stable dose of their antiparkinsonian treatment for at least 4 weeks; (2) no cognitive impairment, defined by Mini-Mental State Examination score ≥  24; (3) Hoehn and Yahr (H&Y) stage [25] between 1 and 3; (4) give their consent to participate in the study. The exclusion criteria were as follows: (1) concomitant neurologic and/or psychiatric diseases; (2) previous use of melatonin supplementation; (3) apnea–hypopnea index and/or periodic limb movements ≥ 15 per hour at PSG; (4) shift workers, who cannot ensure traditional night-time sleep habits; (5) changes in sleep medications or other treatments during the study. Participants were on the same antiparkinsonian medication and did not change any of their medication during the study.

At baseline (T0), before starting the melatonin PR treatment (2 mg), all participants performed a PSG recording and answered subjective sleep questionnaires: PSQI, Parkinson Disease Sleep Scale – 2^nd^ edition (PDSS-2), and Epworth Sleepiness Scale (ESS). Moreover, patients’ motor disability was also assessed using the motor examination section of the Unified Parkinson’s Disease Rating Scale (UPDRS-III). At T0, the following data were also collected from patient medical records: gender, age, disease severity and duration, antiparkinsonian treatment, counted as levodopa equivalent daily dose (LEDD) [26], sleep medication, and antidepressant medication usage. Then, patients were instructed to take melatonin PR (2 mg) 2 hours before going to bed. After 3 months of treatment with melatonin PR (T1), patients repeated the subjective sleep questionnaires, the PSG, and the UPDRS-III. Three different neurologists performed separately the PSG, the subjective sleep questionnaires, and the motor examination to reproduce a single-blind effect. Consistently, neurologists were completely blind to the results of the other examinations.

The study was approved by the local Ethics Committee and was conducted according to the Helsinki Declaration of 1975. All the participants provided their signed informed consent.

### Clinical assessment

At T0, the severity of disease was rated by the H&Y scale, the disease duration was calculated from the time of PD diagnosis, and LEDD was calculated for each participant. Motor disability was scored at T0 and then repeated at T1 (3-month follow-up visit) through the UPDRS-III rated in the morning, before starting the antiparkinsonian treatment [27].

The clinical global improvement (CGI) was used to evaluate the therapeutic response of melatonin on PD patients’ sleep quality [28–30]. The CGI score is obtained through one query rated on a 7-point Likert scale (from 1 = very much improved to 7 = very much worse since the initiation of the treatment) in which the clinician/physician rates the patients’ overall clinical condition after starting treatment. Patients were also asked to report their beliefs about the efficacy of melatonin PR treatment by filling out the self-report patient clinical global impression (PCGI). The PCGI consists of one item rated on a 7-point scale depicting a patient’s rating of overall improvement (1 = very much improved to 7 = very much worse since the initiation of the treatment) [28–31].

### Measures

#### Objective sleep assessment

The effect of melatonin PR on sleep was measured through one-night ambulatory PSG (SOMNOscreen, SOMNOmedics GmbH, Randersacker, Germany) recorded at T0 and then repeated at T1. The signal was stored on a flashcard using a common mean reference and a time constant of 0.3 s. Electrodes were positioned according to the International 10–20 System. The montage consisted of two oculographic channels, three electromyography channels (mental and anterior tibialis muscles), and eight EEG channels (F4, C4, O2, A2, F3, C3, O1, A1). Cardiorespiratory parameters were assessed by recording oronasal flow, thoracic and abdominal movements (plethysmography), pulse oximetry, and electrocardiography. Patients were also instructed to maintain the usual sleep schedule and record it in a sleep diary during the week preceding the evaluation. Sleep analysis was performed according to the standard criteria and the following standard parameters were computed [32]: time in bed (TIB, time spent in bed between lights off and lights on); sleep onset latency (SL, the time-interval between the lights off and the first sleep epoch); total sleep time (TST, the sleep time without SL and awakenings); sleep efficiency (SE, the ratio between TST and TIB); REM sleep latency (LREM, the time-interval between the sleep onset and the first epoch of REM); stage 1 of non-REM sleep (N1); stage 2 of non-REM sleep (N2); stage 3 of non-REM sleep (N3); REM sleep (REM); wakefulness after sleep onset (WASO). Sleep stages percentages were calculated during the TST. The PSG scorers identified apnea/hypopnea events and periodic limb movements based on the international standard criteria of the American Academy of Sleep Medicine [33].

#### Subjective sleep assessments

Subjective sleep complaints were assessed by the PSQI and the PDSS-2 at T0 and T1. The PSQI contains 19 item yielding seven components: subjective sleep quality, sleep latency, sleep duration, sleep efficiency, sleep disturbances, use of sleep medications, and daytime dysfunction [24, 34]. Each component is scored from 0 to 3, and the global PSQI score ranges from 0 and 21, with higher scores indicating poorer quality of sleep. A global PSQI score over 5 is considered as an indicator of relevant sleep disturbances.

The PDSS-2 is a specific and pragmatic clinical tool that considers the multi-factorial nature and the severity of sleep disturbances in PD [35]. The PDSS-2 includes 15 items assessing various sleep and nocturnal disturbances, with a response format from 0 (never) to 4 (always), and a total score ranging from 0 (no disturbances) to 60 (maximum nocturnal disturbance) [35, 36].

Daytime sleepiness was assessed with the ESS [37, 38]. Participants were asked to rate, on a 4-point scale (0–3), their usual chances of dozing off or falling asleep while engaged in eight different activities. ESS score ranges from 0 to 24, with a total score > 9 representing excessive daytime sleepiness.

### Statistical analysis

Statistical analyses were performed using SPSS 25.0 statistical software [39]. Continuous variables are expressed as mean and SD; categorical variables are presented as frequencies and percentages. The mean change in primary and secondary outcome parameters between T0 and T1 was also assessed using the Wilcoxon Rank-Sum test for comparing continuous variables. Moreover, delta change (D) scores between baseline and 3-month follow-up for PSG data, subjective sleep questionnaires, and motor symptoms were calculated. Spearman’s correlation test was used to evaluate the correlation among D scores for PSG data, subjective sleep questionnaires, and motor symptoms and clinical characteristics. All *p*-values lower or equal to 0.05 were considered statistically significant.

## Results

Twelve patients with PD ranging from H&Y stage I to III and under stable antiparkinsonian treatment were included in the study. Males represent most of the sample (83.3%; *n* = 10), and females were two (16.7%). At T0, one patient was taking benzodiazepine medication (8.3%), and two patients were taking anti-depressive drugs (16.7%), which remained stable during the study period. Patients’ demographic and clinical characteristics are presented in Table 1.

**Table 1:** Demographic and clinical information of patients with PD

	PD patients
Males, *n* (%)	10 (83.3%)
Mean age, years	62.83 ± 10.68
Disease duration, years	6.67 ± 5.89
H&Y stage	1.75 ± 0.62
LEDD (mg/day)	500.42 ± 269.35
UPDRS-III	21.50 ± 8.80

Continuous data are presented as mean ± standard deviation. Abbreviations*: H&Y*, Hoehn and Yahr; *LEDD*, levodopa equivalent daily dose; *UPDRS-III*, Unified Parkinson’s Disease Rating Scale

Regarding the objective sleep evaluation, the PSG parameters analysis between T0 and T1 showed that the mean values of SL significantly decreased after 3-month treatment with melatonin-PR, but no other significant differences were found. Notably, at the 3-month follow-up visit, SE improved without reaching statistical significance. All PSG data are reported in Table 2.

**Table 2:** PSG data from baseline to the 3-month follow-up visit

	Baseline (mean ± SD)	3-month FU (mean ± SD)	*p-v*alue
Lights off time (hh:mm:ss PM)	11:10:33 ± 1:10:23	11:14:18 ± 0:56:54	0.386
Time in bed (min)	408.93 ± 84.83	427.99 ± 62.86	0.814
Sleep latency (min)	24.43 ± 45.68	6.13 ± 7.22	0.034
Total sleep time (min)	316.92 ± 74.30	369.92 ± 65.03	0.136
Sleep efficiency (%)	78.56 ± 13.86	86.11 ± 7.03	0.084
REM sleep latency (min)	94.29 ± 46.11	116.96 ± 79.79	0.388
N1 (%)	6.41 ± 3.01	6.74 ± 6.59	0.505
N2 (%)	53.24 ± 10.30	50.10 ± 13.59	0.272
N3 (%)	25.79 ± 12.13	27.98 ± 12.99	0.308
REM (%)	14.53 ± 4.33	15.18 ± 7.20	1.000
WASO (min)	92.01 ± 67.41	58.38 ± 28.31	0.117

Abbreviations: *FU*, follow-up; *WASO*, wakefulness after sleep onset; *N1*, stage 1 of non-REM sleep; *N2*, stage 2 of non-REM sleep; *N3*, stage 3 of non-REM sleep; *REM*, rapid eye movement sleep; *SD*, standard deviation

The analyses of subjective sleep questionnaires, as shown in Table 3, demonstrated that melatonin PR significantly reduced the ESS scores and presented a trend of improvement at the PSQI global scores from T0 to T1. Regarding motor symptoms, a trend in reducing the UPDRS-III score was evident although no significant differences were found between baseline and 3-month follow-up (Table 3).

**Table 3:** Subjective sleep data and motor symptoms from baseline to the 3-month FU visit

	Baseline (mean ± SD)	3-month FU (mean ± SD)	*p*-value
PSQI	8.08 ± 2.81	6.58 ± 2.68	0.057
PDSS-2	16.75 ± 5.91	14.42 ± 5.89	0.239
ESS	8.75 ± 3.65	6.75 ± 2.86	0.026
UPDRS-III	21.50 ± 8.80	20.50 ± 8.24	0.072

Abbreviations: *FU*, follow-up; *PSQI*, Pittsburgh Sleep Quality Index; *PDSS-2*, Parkinson Disease Sleep Scale-Revised; *ESS*, Epworth Sleepiness Scale; *UPDRS-III*, Unified Parkinson’s Disease Rating Scale-III; *SD*, standard deviation

Regarding the PCGI scale, 33.3% of the patients considered that their insomnia symptoms had minimally improved after treatment, 25% answered that it had much improved, 16.7% reported no change, and the remaining 16.7% referred a minimally worsening after the treatment. The evaluation in terms of clinical global improvement change performed by the physician (CGI) was similar to the patients’ perception (Table 4).

**Table 4:** Patients’ and physicians’ clinical global improvement scores for sleep quality

		*N*	%
PCGI	Very much improved	1	8.3
	Much improved	3	25.0
	Minimally improved	4	33.3
	No change	2	16.7
	Minimally worse	2	16.7
	Much worse	0	0
CGI	Very much improved	1	8.3
	Much improved	3	25.0
	Minimally improved	4	33.3
	No change	3	25.0
	Minimally worse	1	8.3
	Much worse	0	0

Abbreviations: *PCGI*, patient clinical global impression; *CGI*, clinical global impression

Finally, the D score from T0 to T1 for PDSS-2 positively correlated with the D score for the REM stage (*rho* = 0.78, *p* < 0.001) and negatively correlated with both the D scores for N2 (*rho* =  − 0.74, *p* < 0.001) and LREM (*rho* =  − 0.63, *p* < 0.05) (Table 5). The D score for PSQI negatively correlated with the D for LREM (*rho* =  − 0.68, *p* < 0.05). The D score for UPDRS-III negatively correlated with the D for SE (*rho* =  − 0.89, *p* < 0.001). PCGI scores were negatively correlated with LEDD (*rho* =  − 0.65, *p* < 0.05), and D scores for LREM (*rho* =  − 0.70, *p* < 0.05) and N2 (*rho* =  − 0.76, *p* < 0.01), and positively correlated with D for PDSS-2 (*rho* = 0.95, *p* < 0.01) and REM (*rho* = 0.71, *p* < 0.01). CGI scores were positively correlated the D score for ESS (*rho* = 0.59, *p* < 0.05), UPDRS-III (*rho* = 0.86, *p* < 0.01), and WASO (*rho* = 0.61, *p* < 0.05). Moreover, CGI scores were negatively correlated with the H&Y stage (*rho* =  − 0.75, *p* < 0.01) and with the D scores for SE (*rho* =  − 0.80, *p* < 0.01).

**Table 5:**
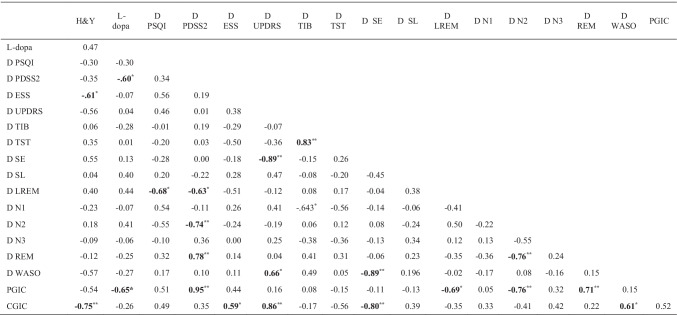
Correlations between clinical characteristics and Delta change mean scores for PSG, subjective sleep questionnaires and motor symptoms

^*^ *p* < 0.05** *p* < 0.001. Note: In bold the significant results. Abbreviations: *H&Y*, Hoehn and Yahr scale; *D*, delta change score; *CGIC*, Clinical Global Improvement; *PGIC*, Patients Clinical Global Improvement; *PSQI*, Pittsburgh Sleep Quality Index; *PDSS-2*, Parkinson Disease Sleep Scale Revised; *ESS*, Epworth Sleepiness Scale; *UPDRS-III*, Unified Parkinson’s Disease Rating Scale-III; *TIB*, time in bed; *TST*, total sleep time; *SE*, sleep efficiency; *SL*, sleep latency; *LREM*, REM latency; *N1*, stage 1 of non-REM sleep; *N2*, stage 2 of non-REM sleep; *N3*, stage 3 of non-REM sleep; *REM*, rapid eye movement sleep; *WASO*, wakefulness after sleep onset

## Discussion

Sleep-related symptoms, especially insomnia, are frequently reported by patients with PD, impair their well-being, influence their daily living activities, and aggravate motor functioning [2]. In support of the importance of maintaining sleep quality and continuity in patients with PD, the concept of sleep benefit has been established several years ago. Sleep benefit can ameliorate patients’ QoL [40, 41], but its main effect is the reduction of motor impairment and the improvement in levodopa response. Although clinicians should target sleep benefit in patients with PD, currently available pharmacological or non-pharmacological strategies failed to ensure this positive effect [42].

Among the different approaches for sleep disorders, melatonin has been reported to produce beneficial effects on sleep-related symptoms and non-motor symptoms in PD [14, 15, 21, 43]. Pointedly, animal studies proved that melatonin may improve PD-related neurodegenerative processes, including dopamine cell loss, neuroinflammation, and alpha-synuclein pathology [16–20]. Moreover, both research studies involving humans and clinical trials in patients with PD reported that melatonin or melatonin PR significantly improves subjective sleep quality [14, 15, 21, 22], prolongs total sleep time measured by actigraphy [21], and reduces the burden of NMS [14, 15, 22]. Despite these few studies have shown that melatonin improves the quality of sleep, the effects on motor symptoms are still not clear since the improvement of UPDRS-III scores was not documented [15, 22]. To further explore these findings and recognize the importance of targeting sleep benefit for improving PD motor and non-motor symptoms, this observational pilot study preliminary investigated the effects of melatonin PR on subjective and objective sleep related-symptoms and motor disability in a representative sample of PD patients.

The main results of the present study are the objective improvement in sleep quality, as documented by the reduction of SL, combined with the subjective improvement of daytime sleepiness, measured through ESS, a validated instrument currently used in PD patients [44, 45], after 3 months of melatonin 2 mg PR treatment. Although not achieving the statistical significance, probably due to the small sample of patients included, a clear trend in improving SE, estimated through the PSG recording, was evident. The PSQI also presents a clear trend in reducing the total score at follow-up thus reflecting a better objective and subjective sleep quality. Notably, motor impairment evaluated the morning after the PSG recording, before anti-PD treatment administration, also presented a trend in amelioration, since scores at the UPDRS-III reduced after 3 months of melatonin 2 mg PR treatment. Combining the motor and sleep data, the documentation of a significant correlation between the improvement in SE and the reduction of UPDRS-III scores after the 3 months of treatment supports the importance of targeting sleep benefit in patients with PD.

As previously stated, melatonin treatment has been already suggested in patients with PD since its beneficial effects on both neurodegenerative processes and PD symptoms. The dysregulation of the pituitary gland and melatonin secretion has been demonstrated in PD. In particular, Videnovic and colleagues documented that the melatonin circadian rhythmicity is altered in PD patients, contributing to their excessive daytime sleepiness [46]. The main dysregulation in circadian melatonin secretion was the reduced amplitude of the melatonin rhythm and its circulating levels [46], possibly due to the dysfunction of the suprachiasmatic nucleus (SCN) and its afferent and efferent pathways. Moreover, an autoptic study documented the impairment of SCN in patients with in vivo diagnosis of PD, which showed the typical PD neuropathological features [47]. These findings lead to the hypothesis that the SCN and pituitary dysfunction seem to play a role in the multi-faced etiopathology of sleep impairment in PD patients.

Therefore, the present findings, although achieved in a small sample of patients, propose melatonin PR as a beneficial treatment for sleep-related symptoms in PD patients, who can improve their motor and sleep-related PD symptoms through the sleep benefit effect, without changing antiparkinsonian treatment. Moreover, the significant reduction of daytime sleepiness after melatonin PR treatment can be also related to the restoration of the circadian melatonin secretion, which is altered in PD patients and can not be completely related to sleep impairment [48].

The present study presents some limitations. First, the lack of a control group and the single-blinded observation requires a double-blind placebo-controlled trial to confirm this preliminary observation, although PSG raters were blinded to the subjective sleep data and to patients’ perception of sleep. Second, considering the nature of this pilot investigation, more studies are needed to confirm these findings, also contemplating wider sample sizes. In fact, the number of participants for this study is small and thus prevents the generalization of the results. Third, a quarter of patients were currently taking benzodiazepine or antidepressant drugs, which may have affected the current results, although all the treatments remained unchanged during the study period.

Although we are aware of the limitations of the present study, this study suggests the clinical potential of using melatonin 2 mg PR in PD patients to improve sleep quality, excessive daytime sleepiness, and possibly to reduce motor symptoms through the sleep benefit effect. Moreover, the improvement in sleep quality and motor symptoms was more evident in patients with lower LEDD doses and at lower H&Y stages. This finding opens the possibility to early identify sleep problems to reduce motor impairment, and also the LEDD in patients with PD. However, our results should be considered with caution since their preliminary nature.

In conclusion, although the exact mechanism to increase melatonin function and activity remains unclear, there is a growing interest in melatonin as a potential therapeutic agent in neurodegenerative disorders, including Alzheimer’s disease and PD. Therefore, melatonin presents itself as a safe therapeutic option for sleep disorders [14, 49, 50], and the potentiality of a supplementation regimen with both melatonin and melatonin PR should be considered in PD patients for obtaining the sleep benefit on motor and non-motor symptoms.

## Data Availability

The data that support the findings of this study are available from the corresponding author upon reasonable request.
